# The association between preoperative body composition and aerobic fitness in patients scheduled for colorectal surgery

**DOI:** 10.1111/codi.15941

**Published:** 2021-10-20

**Authors:** Annefleur E. M. Berkel, Laura van Wijk, David P. J. van Dijk, Sanne N. Prins, Job van der Palen, Nico L. U. van Meeteren, Steven W. M. Olde Damink, Joost M. Klaase, Bart C. Bongers

**Affiliations:** ^1^ Department of Surgery Medisch Spectrum Twente Enschede The Netherlands; ^2^ Department of Hepatobiliary Surgery and Liver Transplantation University Medical Center Groningen Groningen The Netherlands; ^3^ Department of Surgery NUTRIM School of Nutrition and Translational Research in Metabolism Maastricht University Maastricht The Netherlands; ^4^ Medical School Twente Medisch Spectrum Twente Enschede The Netherlands; ^5^ Department of Research Methodology Measurement and Data Analysis University of Twente Enschede The Netherlands; ^6^ Top Sector Life Sciences and Health (Health~Holland) The Hague The Netherlands; ^7^ Department of Anesthesiology Erasmus Medical Center Rotterdam The Netherlands; ^8^ Department of Epidemiology Care and Public Health Research Institute (CAPHRI) Maastricht University Maastricht The Netherlands; ^9^ Department of Nutrition and Movement Sciences NUTRIM School of Nutrition and Translational Research in Metabolism Maastricht University Maastricht The Netherlands

**Keywords:** anaerobic threshold, cardiopulmonary exercise test, physical fitness, prehabilitation, preoperative risk assessment, skeletal muscle mass

## Abstract

**Aim:**

Although cardiopulmonary exercise testing (CPET) is considered the gold standard, a preoperative abdominal CT scan might also provide information concerning preoperative aerobic fitness for risk assessment. This study aimed to investigate the association between preoperative CT‐scan‐derived body composition variables and preoperative CPET variables of aerobic fitness in colorectal surgery.

**Method:**

In this retrospective cohort study, CT images at level L3 were analysed for skeletal muscle mass, skeletal muscle radiation attenuation, visceral adipose tissue (VAT) mass and subcutaneous adipose tissue mass. Regression analyses were performed to investigate the relation between CT‐scan‐derived body composition variables, CPET‐derived aerobic fitness and other preoperative patient‐related variables. Logistic regression analysis was performed to predict a preoperative anaerobic threshold (AT) ≤ 11.1 ml/kg/min as cut‐off for having a high risk for postoperative complications.

**Results:**

Data from 78 patients (45 men; mean [SD] age 74.5 [6.4 years]) were analysed. A correlation coefficient of 0.55 was observed between absolute AT and skeletal muscle mass index. Absolute AT (*R* ^2^ of 51.1%) was lower in patients with a lower skeletal muscle mass index, together with higher age, lower body mass and higher American Society of Anesthesiologists (ASA) score. Higher ASA score (odds ratio 5.64; *P* = 0.033) and higher VAT mass (odds ratio 1.02; *P* = 0.036) were associated with an increased risk of an AT ≤ 11.1 ml/kg/min.

**Conclusion:**

Body composition variables from the preoperative CT scan were moderately associated with preoperative CPET‐derived aerobic fitness. Higher ASA score and higher VAT mass were associated with an increased risk of an AT ≤ 11.1 ml/kg/min.

**What does this paper add to the literature?**

Although cardiopulmonary exercise testing (CPET) is the gold standard test to assess aerobic fitness, it is not always available in clinical practice. From this study, it appears that the routinely performed preoperative abdominal computed tomography scan cannot replace CPET for preoperative risk assessment on aerobic fitness in colorectal surgery.

## INTRODUCTION

Colorectal cancer is the third most common type of cancer [1]. After resection for colon or rectal carcinoma, 15% and 20% of the patients respectively have a complicated course within 30 days after surgery, which might lead to a prolonged hospital stay of >14 days or even mortality [2]. Reducing complications will result in considerable cost savings [3]. Preoperative risk assessment might identify patients at high risk of postoperative complications; these patients may benefit from preoperative preventive interventions (prehabilitation) [4, 5].

Cardiopulmonary exercise testing (CPET) is increasingly utilized for risk assessment before major surgery to evaluate the risk of adverse perioperative events [5]. CPET is an objective and precise method of evaluating a patient's preoperative aerobic fitness. In general, patients with a lower oxygen uptake at the anaerobic threshold (AT) and/or a lower oxygen uptake at peak exercise (VO_2peak_) have an increased risk of postoperative complications [6, 7, 8, 9]. Despite its usefulness in perioperative medicine, CPET is not always available in clinical practice, is relatively expensive and time‐consuming, and requires well‐trained personnel for an adequate interpretation of its results.

For preoperative risk assessment, measurements of body composition using the routinely performed abdominal CT scan are increasingly gaining ground. Sarcopenia [10], a low skeletal muscle radiation attenuation (SM‐RA) [11, 12] and a high visceral adipose tissue (VAT) mass [13, 14] have all been reported to be associated with poor clinical outcome following abdominal surgery. Furthermore, Boo and others [15] demonstrated that skeletal muscle mass is closely associated with aerobic fitness (the AT and VO_2peak_) in community‐dwelling elderly men, while a recent study by West and others [16] in patients undergoing hepatopancreatobiliary surgery reported that SM‐RA and not skeletal muscle mass (assessed by a preoperative CT scan) were associated with aerobic fitness (assessed with preoperative CPET).

Although CPET is the gold standard to assess aerobic fitness, it would be of interest for time and cost savings to investigate whether the routinely performed preoperative abdominal CT scan can (assist to pre)select unfit patients. Therefore, the aim of this study was to preoperatively investigate the association between body composition variables derived from the abdominal CT scan and CPET variables of aerobic fitness in patients scheduled for colorectal surgery.

## METHOD

The present retrospective study was reported according to the STrengthening the Reporting of OBservational studies in Epidemiology (STROBE) guideline.

### Participants

Data from all patients ≥60 years old with colorectal cancer or dysplasia planned for elective colorectal resection at the hospital Medisch Spectrum Twente, with a veterans‐specific activity questionnaire (VSAQ) score ≤ 7 metabolic equivalents of task (METs) and who underwent a preoperative abdominal CT scan and preoperative CPET between February 2013 and May 2017 were included. The VSAQ is a brief self‐administered questionnaire to estimate aerobic fitness, in which a score ≤ 7 METs was used to preselect those patients with a low perceived aerobic fitness [17]. These formed the study data and were retrospectively analysed after this period. Ethical approval for the study protocol (registration number P13‐18) was provided by the Medical Ethics Committee Twente (Dr J.F.F. Lekkerkerker, Clinical Pharmacologist, chairman) in September 2013, and written informed consent was obtained from each participant. Patients were excluded if the time between CPET and CT was >60 days, or when acute surgery of the tumour was necessary.

### Computed tomography scan

A single slice of each patient's routinely performed preoperative abdominal CT scan was selected at the level of the third lumbar vertebra (L3) on which both transverse processes were visible. CT scans were all screened for their quality. Patients with a CT scan of poor quality (e.g., large radiation artefacts, low dose) were excluded from analysis. Scans were analysed using sliceOmatic 5 (TomoVision) software for Microsoft Windows®. The cross‐sectional areas (cm^2^) of skeletal muscle tissue, VAT and subcutaneous adipose tissue (SAT) were coloured automatically, and manually corrected if necessary, by two trained and blinded researchers (LvW and checked by DvD, both blinded for CPET analyses). Skeletal muscle tissue, VAT and SAT areas were normalized for the patient's body height to calculate the L3 index (cm^2^/m^2^). The SM‐RA was assessed by calculating the average Hounsfield units (HU) value of skeletal muscle mass. Low SM‐RA is associated with increased intermyocellular and intramyocellular fat (myosteatosis) [18].

### Cardiopulmonary exercise testing

As part of the study protocol, an incremental CPET was performed by patients preoperatively under controlled conditions at the lung function department, using a calibrated electronically braked cycle ergometer in upright position (Ergoline, Ergoselect 100). The following standardized pre‐test instructions were given to the patients: (1) consume the last (light) meal at least 2 h before exercise testing, (2) adhere to usual use of medication and (3) wear comfortable sporting clothes and shoes. CPET comprised a 2‐min resting phase to assess baseline cardiopulmonary values, followed by 3 min of unloaded cycling (warm‐up), after which the work rate was progressively increased with constant increments of 5, 10 or 15 W/min, depending on the patient's subjective physical fitness level and aimed at reaching a maximal effort within 8–12 min. Throughout CPET, patients had to maintain a pedalling frequency between 60 and 80 revolutions/min. The protocol continued until the patient's pedalling frequency fell definitely <60 revolutions/min, despite strong verbal encouragement. After test termination, the patient completed a 5‐min recovery phase of unloaded cycling (cool‐down).

During CPET, patients breathed through a facemask (Hans Rudolph) connected to a Triple V volume transducer to calculate breath‐by‐breath minute ventilation, oxygen uptake (VO_2_), carbon dioxide production (VCO_2_) and the respiratory exchange ratio averaged at 10‐s intervals (Oxycon Pro, Jaeger). Flow volume (3‐L syringe) and gas calibration (ambient air and a gas mixture of 16% oxygen and 5% carbon dioxide) were performed manually before each test. Heart rate (HR), 12‐lead electrocardiography, blood pressure and pulse oximetry were continuously monitored.

CPET data were interpreted by a trained and experienced clinical exercise physiologist (BB, blinded for CT scan analyses). The highest HR achieved during the CPET was defined as HR_peak_. Data from other outcome variables were averaged over 30 s of exercise. VO_2peak_ values were considered valid when at least one of the following criteria was met: an HR at peak exercise >95% of predicted (predicted peak HR [beats/min] = 208–0.7 × age [years]) or a respiratory exchange ratio at peak exercise >1.10. The AT was defined as the point at which the ventilatory equivalent for oxygen and the partial end‐tidal oxygen tension reached a minimum and thereafter began to rise in a consistent manner, coinciding with an unchanged ventilatory equivalent for carbon dioxide and partial end‐tidal carbon dioxide tension [19]. If this ventilatory equivalents method provided uncertain results, the V‐slope method was used to estimate the AT (the point at which the linear slope of the relation between the VCO_2_ and VO_2_ changed) [20]. Finally, the oxygen uptake efficiency slope (OUES) which provides a valid objective effort‐independent measure of aerobic fitness in elderly patients scheduled for major colorectal surgery was calculated [21]. Absolute VO_2peak_, AT and OUES values were normalized for body mass as well.

### Patient characteristics and outcome measures

Baseline patient characteristics included sex, age, body height, body mass, body mass index (BMI), smoking status, use of beta‐blocker, METs score on the VSAQ, clinical signs of metastasis, American Society of Anesthesiologists (ASA) score (I–IV) and Charlson comorbidity index (divided into three groups: 0, 1 and 2+). Body composition and aerobic fitness outcomes were reported separately for men and women, as it is known that values significantly differ between sexes.

### Statistical analysis

Data were analysed with the Statistical Package for the Social Sciences for Windows (version 23.0; IBM, SPSS Inc.). Continuous data were presented as mean and standard deviation or as median and interquartile range where appropriate. Categorical data were summarized by frequency and percentage. Pearson or Spearman correlation coefficients were calculated to examine univariable associations between continuous variables, depending upon the distribution of the variables. To investigate the univariable association between a continuous variable (e.g., AT) and a categorical variable, one‐way ANOVA, the independent samples *t* test or the Mann–Whitney *U* test, as appropriate, was used. Univariable associations with a *P* < 0.10 were included in the multivariable analysis. For predicting continuous outcomes, linear regression analyses (method: enter) were performed to investigate the association between continuous CPET variables (dependent variable, e.g., AT) and preoperative independent variables.

A multivariable logistic regression analysis was performed to predict whether a patient had a relative AT ≤ 11.1 ml/kg/min. Preoperative variables were tested for their association with a relative AT ≤ 11.1 ml/kg/min (*P* < 0.10), using the *t* test, Mann–Whitney *U* test, Fisher's exact test or chi‐squared test, as appropriate. A logistic regression model was performed to select which of the remaining variables were significant in a forward stepwise procedure (*P* in 0.10, *P* out 0.15). In the case of multicollinearity between variables, the variable that produced the best model fit (based on the −2 log likelihood) was included in the model. With the final selected significant variables, a new logistic regression model was made (method: enter) to utilize the maximum number of observations. Receiver operating characteristic (ROC) analysis was used to assess the independent ability of predictive variables to discriminate between patients with and without a relative AT ≤11.1 ml/kg/min; this AT cut‐off was based on the work by West and others [8] in patients undergoing major colorectal surgery. The optimal cut‐off point from the ROC analysis was based on our preference to have primarily a high sensitivity (with a reasonable specificity), as we aim to detect almost all high‐risk patients that might benefit from a preoperative intervention (e.g., prehabilitation). A *P* < 0.05 was considered statistically significant.

## RESULTS

### Patients

Between February 2013 and May 2017, a total of 371 potential patients ≥60 years old with a colorectal tumour were assessed for eligibility. Of these patients, 189 (50.9%) had a VSAQ score ≤ 7 METs, of which 91 patients (48.1%) underwent a preoperative CPET.

Of these 91 patients, 13 patients were excluded: in two patients (2.2%) SM‐RA could not be measured using their CT scan; in nine patients (9.9%) raw preoperative CPET data were not available; and in two patients (2.2%) the AT and VO_2peak_ could not be determined due to a poor effort at the CPET (invalid test). Patient characteristics of the remaining 78 patients (45 men and 33 women, mean age 74.5 ± 6.4 SD years, range 61.5–90.3 years) are presented in Table 1.

**TABLE 1:** Patient characteristics

Parameter	Total (*n* = 78)
Age (years)	74.5 ± 6.4
Sex (men)	45 (57.7)
Body height (cm)	169.9 ± 9.3
Men	175.1 ± 7.1
Women	163.0 ± 7.2
Body mass (kg)	84.5 ± 14.3
Men	89.0 ± 13.7
Women	78.5 ± 12.9
Body mass index (kg/m^2^)	29.2 ± 3.8
Men	29.0 ± 3.8
Women	29.5 ± 3.9
Smoking^a^	11 (15.7)
VSAQ score (METs)^b^	5 ± 1
Charlson comorbidity index	
0	23 (29.5)
1	27 (34.6)
≥2	28 (35.9)
ASA score	
I and II	61 (78.2)
III and IV	17 (21.8)
Tumour localization	
Ascending colon	29 (37.2)
Transverse colon	7 (9.0)
Descending colon	5 (6.4)
Sigmoid	23 (29.5)
Rectum^c^	11 (14.1)
Other^d^	3 (3.8)
Clinical metastasis category	
cM0	67 (85.9)
cM1	5 (6.4)
Not applicable^e^	6 (7.7)

Note

Values are presented as mean ± SD or as n (%).

Abbreviations: ASA, American Society of Anesthesiologists; MET, metabolic equivalent of task; VSAQ, veterans‐specific activity questionnaire.

^a^Eight missing values.

^b^Thirteen missing values.

^c^Four patients with a rectal tumour received neoadjuvant chemoradiation; one patient received neoadjuvant radiotherapy.

^d^Two patients had a tumour in both the ascending and transverse colon; one patient had metachronous colorectal liver metastasis.

^e^Includes dysplasia (*n* = 5) and metachronous colorectal liver metastasis (*n* = 1).

All 78 patients performed the CPET without any complications or adverse events during or after the test. The AT was indeterminable in two (2.6%) patients, while they attained a valid VO_2peak_. Normalized for body mass, mean ± SD values of VO_2peak_ and AT were 15.6 ± 3.7 ml/kg/min and 10.6 ± 1.9 ml/kg/min, respectively. Mean ± SD time between the CT scan and CPET was 15.2 ± 15.3 days. CPET results are shown in Table 2.

**TABLE 2:** Preoperative body composition parameters derived from the abdominal CT scan and preoperative CPET parameters

Parameter	Total (*n* = 78)	Men (*n* = 45)	Women (*n* = 33)	*P* value^f^
CT scan parameters				
Skeletal muscle mass index (cm^2^/m^2^)	44.9 ± 11.9	50.9 ± 10.6	36.6 ± 8.1	<0.001
SM‐RA (HU)	29.1 ± 7.6	30.3 ± 7.8	27.5 ± 7.2	0.110
VAT mass (cm^2^/m^2^)	77.8 ± 38.2	86.3 ± 37.9	66.2 ± 36.1	0.021
SAT mass (cm^2^/m^2^)	80.0 ± 30.4	65.2 ± 26.5	100.1 ± 22.9	<0.001
CPET parameters				
HR_peak_ (beats/min)^a^	129 ± 19	128 ± 19	130 ± 19	0.751
Without beta blocker^b^	135 ± 17	137 ± 15	133 ± 20	0.429
With beta blocker^b^	120 ± 18	119 ± 19	122 ± 18	0.728
RER_peak_	1.14 ± 0.11	1.16 ± 0.10	1.12 ± 0.11	0.059
WR_peak_ (W)	98 ± 32	110 ± 32	83 ± 25	<0.001
WR_peak_ (W/kg)	1.2 ± 0.3	1.2 ± 0.3	1.1 ± 0.3	0.030
VO_2peak_ (ml/min)	1312 ± 351	1413 ± 348	1173 ± 309	0.002
VO_2peak_ (ml/kg/min)	15.6 ± 3.7	16.0 ± 3.8	15.1 ± 3.5	0.262
AT (ml/min)^c^	889 ± 181	937 ± 175	824 ± 171	0.006
AT (ml/kg/min)^c^	10.6 ± 1.9	10.6 ± 1.9	10.5 ± 1.7	0.823
O_2_ pulse_peak_ (ml/beat) ^a^	10.3 ± 2.6	11.2 ± 2.7	9.0 ± 2.0	<0.001
O_2_ pulse_peak_ (ml/kg/beat × 100)^a,d^	12.3 ± 2.3	12.8 ± 2.6	11.7 ± 1.9	0.056
VE/VCO_2_ slope^e^	33.2 ± 6.6	33.8 ± 7.8	32.4 ± 4.6	0.375
VE_peak_ (l/min)	56.6 ± 17.0	62.5 ± 16.7	48.7 ± 14.1	<0.001
VE_peak_ (l/kg/min)	0.7 ± 0.2	0.7 ± 0.2	0.6 ± 0.2	0.094
OUES	1576 ± 444	1695 ± 428	1413 ± 418	0.005
OUES/kg	18.7 ± 4.5	19.2 ± 4.7	18.0 ± 4.2	0.248

Note

Values are presented as mean ± SD.

Abbreviations: AT, anaerobic threshold; CPET, cardiopulmonary exercise testing; HR_peak_, heart rate at peak exercise; HU, Hounsfield units; O_2_ pulse_peak_, oxygen pulse at peak exercise; OUES, oxygen uptake efficiency slope; RER_peak_, respiratory exchange ratio at peak exercise; SAT, subcutaneous adipose tissue; SM‐RA, skeletal muscle radiation attenuation; VAT, visceral adipose tissue; VE/VCO_2_ slope, minute ventilation to carbon dioxide production relationship; VE_peak_, minute ventilation at peak exercise; VO_2peak_, oxygen uptake at peak exercise; WR_peak_, work rate at peak exercise.

^a^Heart rate was invalid in eight patients (10.3%, six men and two women), so in this case *n* = 70.

^b^A beta‐blocker was used by 26 patients (17 men and nine women), 43 patients did not use a beta blocker, and in one patient beta blocker use was unknown.

^c^The AT was not determinable in two patients (2.6%, one man and one woman), so in this case *n* = 76.

^d^O_2_ pulse values normalized for body mass are multiplied by 100 to increase readability.

^e^The VE/VCO_2_ slope was calculated using data up to the respiratory compensation point.

^f^Independent samples *t* tests.

Mean ± SD skeletal muscle mass index was 50.9 ± 10.6 cm^2^/m^2^ in men (range 31.1–91.5) and 36.6 ± 8.1 cm^2^/m^2^ in women (range 20.4–66.7). CT scan measurements are depicted in Table 2.

### Association between preoperative body composition parameters derived from the abdominal CT scan and preoperative CPET parameters

In the univariable analysis (Table 3), a Pearson correlation coefficient of 0.55 (*P* < 0.001) was found between the absolute AT and skeletal muscle mass index. Between the relative AT and skeletal muscle mass index, a correlation coefficient of 0.16 (*P* = 0.156) was observed. A Pearson correlation coefficient of 0.28 (*P* = 0.014) was found between the relative AT and SM‐RA.

**TABLE 3:** Correlation coefficients between preoperative body composition parameters derived from the abdominal CT scan and preoperative CPET parameters

Parameter	Skeletal muscle mass index (cm^2^/m^2^)	SM‐RA (HU)	VAT mass (cm^2^/m^2^)	SAT mass (cm^2^/m^2^)
AT (ml/min)^a^	0.55 (*P* < 0.001)	0.08 (*P* = 0.472)	0.22 (*P* = 0.063)	0.03 (*P* = 0.783)
AT (ml/kg/min)^a^	0.16 (*P* = 0.156)	0.28 (*P *= 0.014)	−0.24 (*P* = 0.040)	−0.16 (*P* = 0.177)
VO_2peak_ (ml/min)	0.51 (*P* < 0.001)	0.10 (*P* = 0.369)	0.18 (*P* = 0.122)	−0.09 (*P* = 0.427)
VO_2peak_ (ml/kg/min)	0.22 (*P* = 0.058)	0.26 (*P *= 0.020)	−0.17 (*P* = 0.130)	−0.24 (*P* = 0.034)
VE/VCO_2_ slope^b^	−0.12 (*P* = 0.281)	−0.17 (*P* = 0.127)	−0.02 (*P* = 0.889)	−0.10 (*P* = 0.390)
OUES	0.40 (*P* < 0.001)	<−0.01 (*P* = 0.991)	0.23 (*P* = 0.045)	<−0.01 (*P* = 0.979)
OUES/kg	0.13 (*P* = 0.246)	0.15 (*P *= 0.202)	−0.12 (*P* = 0.287)	−0.18 (*P* = 0.120)

Abbreviations: AT, anaerobic threshold; CPET, cardiopulmonary exercise testing; HU, Hounsfield units; OUES, oxygen uptake efficiency slope; SM‐RA, skeletal muscle radiation attenuation; SAT, subcutaneous adipose tissue; VAT, visceral adipose tissue; VE/VCO_2_ slope, minute ventilation to carbon dioxide production relationship; VO_2peak_, oxygen uptake at peak exercise.

^a^The AT was not determinable in two patients (2.6%, one man and one woman), so in this case *n* = 76.

^b^The VE/VCO_2_ slope was calculated using data up to the respiratory compensation point.

Variables with a *P* < 0.10 in the univariable analysis (age, body mass, body height, ASA, sex, skeletal muscle mass index and VAT mass) were included in a multivariable linear regression analysis to predict the absolute AT. BMI was also associated with absolute AT (*P* < 0.10) but was not included in the multivariable analysis because of multicollinearity between BMI, body mass and body height. In the final multivariable model (*R* ^2^ 51.1%), a lower age, a higher body mass, a lower ASA score and a higher skeletal muscle mass index were associated with a higher absolute AT (Table 4):

$$\text{Absolute}\;\text{AT}\;\left(\text{ml}/\text{min}\right)=848.6‐\left(4.99\times \text{age}\;\text{in}\;\text{years}\right)+\left(4.18\times \text{body}\;\text{mass}\;\text{in}\;\text{kg}\right)‐\left(124.4\times \text{ASA}\;\text{score}\right)+\left(4.65\times \text{skeletal}\;\text{muscle}\;\text{mass}\;\text{index}\;\text{in}\;{\text{cm}}^{2}/m^{2}\right)$$

**TABLE 4:** Multivariable linear regression analysis to predict the preoperative absolute and relative AT and absolute and relative VO_2peak_

Predicted CPET variable	Parameter	*B*	95% CI	*P* value
Absolute AT (ml/min)	Age (years)	−5.00	−9.80–−0.19	0.042
	Body mass (kg)	4.18	1.69–6.66	0.001
	ASA score	−124	−199–−49.8	0.001
	Skeletal muscle mass index (cm^2^/m^2^)	4.65	1.69–7.62	0.003
Relative AT (ml/kg/min)	Body mass index (kg/m^2^)	−0.13	−0.23–−0.03	0.014
	ASA score	−1.80	−2.70–−0.90	<0.001
	SM‐RA (HU)	0.05	−0.004–0.10	0.071
Absolute VO_2peak_^a^ (ml/min)	Age (years)	−12.0	−21.3–−2.63	0.013
	Body height (cm)	12.5	5.34–19.7	0.001
	ASA score	−270	−413–−128	<0.001
	Skeletal muscle mass index (cm^2^/m^2^)	8.22	2.69–13.8	0.004
Relative VO_2peak_^b^ (ml/kg/min)	Age (years)	−0.14	−0.24–−0.04	0.008
	Body mass index (kg/m^2^)	−0.42	−0.59–−0.25	<0.001
	ASA score	−2.40	−4.11–−0.69	0.007
	Charlson comorbidity index	−1.12	−1.98–−0.26	0.012
	Skeletal muscle mass index (cm^2^/m^2^)	0.09	0.03–0.15	0.003

Abbreviations: ASA, American Society of Anesthesiologists; AT, anaerobic threshold; BMI, body mass index; HU, Hounsfield units; SM‐RA, skeletal muscle radiation attenuation; VO_2peak_, oxygen uptake at peak exercise.

^a^In a formula, absolute VO_2peak_ (ml/min) = 34.9 – (12.0 × age in years) + (12.5 × body height in cm) – (270 × ASA score) + (8.22 × skeletal muscle mass index in cm^2^/m^2^). For an ASA score 1 or 2, a 1 must be used, whereas for an ASA score 3 or 4 a 2 should be used in the equation.

^b^In a formula, relative VO_2peak_ (ml/kg/min) = 38.4 – (0.14 × age in years) – (0.42 × BMI in kg/m^2^) – (2.40 × ASA score) – (1.12 × Charlson score) + (0.09 × skeletal muscle mass index in cm^2^/m^2^). For an ASA score 1 or 2, a 1 must be used, whereas for an ASA score 3 or 4 a 2 should be used in the equation. For a Charlson score 0, a 0 should be used; for a Charlson score 1, a 1 must be used; and for a Charlson score 2+, a 2 should be used in the equation.

For an ASA score of 1 or 2, a 1 must be used, whereas for an ASA score of 3 or 4 a 2 should be used in the equation.

Moreover, variables with a *P* < 0.10 in the univariable analysis (BMI, ASA, VSAQ score, SM‐RA and VAT mass) were included in the multivariable linear regression analysis to predict the relative AT. Body mass was also associated with relative AT (*P* < 0.10) but was not included in the multivariable analysis because of multicollinearity between body mass and BMI. In the final multivariable model (*R* ^2^ 28.6%), a higher BMI, a higher ASA score and a lower SM‐RA were associated with a lower relative AT (Table 4):

$$\text{Relative}\;\text{AT}\;\left(\text{ml}/\text{kg}/\text{min}\right)=15.1‐\left(0.13\times \text{BMI}\;\text{in}\;\text{kg}/m^{2}\right)‐\left(1.80\times \text{ASA}\;\text{score}\right)+\left(0.05\times \text{SM - RA}\right)$$

For an ASA score of 1 or 2, a 1 must be used, whereas for an ASA score of 3 or 4 a 2 should be used in the formula. The multivariable linear regression analyses to predict the absolute and relative VO_2peak_ can be found in Table 4.

### Prediction of a preoperative relative anaerobic threshold ≤11.1 ml/kg/min

A multivariable logistic regression analysis was performed to investigate if a preoperative relative AT ≤ 11.1 ml/kg/min can be predicted from body composition variables derived from the abdominal CT scan and other patient characteristics. In the univariable analysis, age, body mass, BMI, VAT mass, ASA score, VSAQ score and Charlson score were associated with a relative AT ≤ 11.1 ml/kg/min (with a *P* < 0.10) and were included in a forward stepwise multivariable analysis. A higher ASA score (OR 6.95, 95% CI 0.81–59.3, *P* = 0.076) and a higher VAT mass (OR 1.01, 95% CI 1.00–1.03, *P* = 0.090) were associated with an increased risk of a relative AT ≤ 11.1 ml/kg/min. Another logistic regression model was made (method: enter), with ASA and VAT mass, to include all patients (as, although ≤ 7 METs, the exact VSAQ scores of 13 patients were missing). In this final model, a higher ASA score (OR 5.64, 95% CI 1.15–27.7, *P* = 0.033) and a higher VAT mass (OR 1.02, 95% CI 1.00–1.03, *P* = 0.036) were associated with an increased risk of a relative AT ≤ 11.1 ml/kg/min. Patients with an ASA score of 3 or 4 were almost six times more likely to have a relative AT ≤ 11.1 ml/kg/min.

**FIGURE 1:**
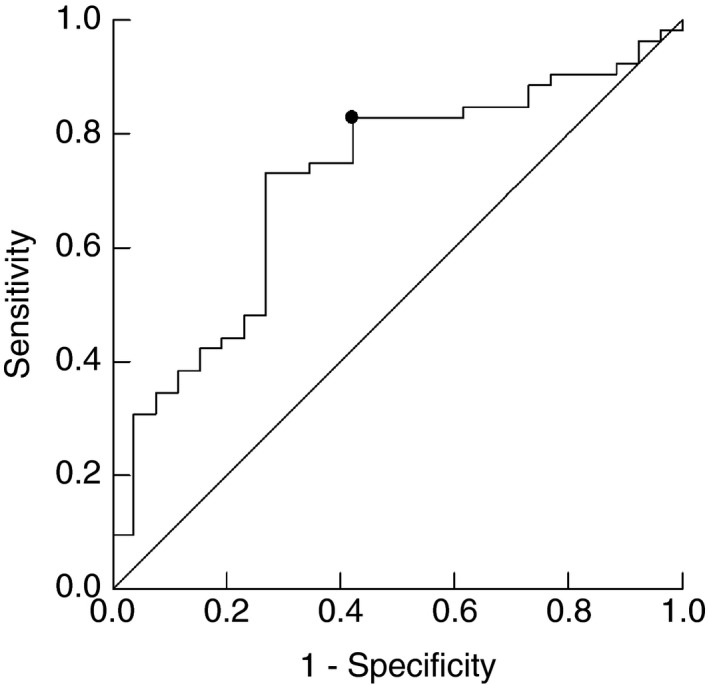
ROC analysis for predicting patients with a relative AT ≤ 11.1 ml/kg/min from the ASA score and visceral adipose tissue (AUC 0.71; 95% CI 0.60–0.83; *P* = 0.002). Abbreviations: ASA, American Society of Anesthesiologists; AT, anaerobic threshold; AUC, area under the curve; ROC, receiver operating characteristic

ROC analysis for predicting patients with a relative AT ≤ 11.1 ml/kg/min from ASA score and VAT mass gave an area under the curve (AUC) of 0.71 (95% CI 0.60–0.83, *P* = 0.002) (Figure 1). Patients with a relative AT ≤ 11.1 ml/kg/min can be predicted with the formula 1/(1 + exp{−[−0.74 + (0.02 × VAT mass) + (1.73 × ASA)]}). For an ASA score of 1 or 2, a 0 must be used, whereas for an ASA score of 3 or 4 a 1 should be used in the equation. When choosing a cut‐off point of 0.55, the sensitivity was 82.7% and specificity was 46.2%, while the positive predictive value was 75.4% and the negative predictive value was 57.1%.

## DISCUSSION

This study aimed to investigate the association between body composition variables derived from the preoperative abdominal CT scan and preoperative CPET variables of aerobic fitness in patients scheduled for colorectal surgery, to evaluate whether the preoperative CT scan can (assist to pre)select unfit patients. The results demonstrated that body composition variables were significantly associated with preoperative aerobic fitness, expressed as the absolute and relative AT, absolute and relative VO_2peak_ and OUES. In the multivariable regression model to predict the preoperative absolute AT, it was found that the absolute AT (*R* ^2^ 51.1%) was lower in patients with a lower skeletal muscle mass index, together with a higher age, a lower body mass and a higher ASA score. Variation in relative AT values (*R* ^2^ 28.6%) could be less well explained by body composition variables and other patient‐related variables.

Body composition variables such as skeletal muscle mass correlate better with absolute measures of aerobic fitness (AT, VO_2peak_ and OUES) than with relative variables (here normalized for body mass) of aerobic fitness. This can be explained by the fact that skeletal muscle mass represents an absolute measure of the body's skeletal muscle mass, and a higher absolute skeletal muscle mass generally results in greater exercise‐induced peripheral oxygen extraction and utilization by the exercising muscles, which is an important determinant for absolute aerobic fitness. Aerobic fitness refers to the maximal capacity of the pulmonary and cardiovascular system to take in and transport oxygen to the exercising muscles, and of those exercising muscles to extract and utilize oxygen from the blood for aerobic respiration [22]. Thus, aerobic fitness depends not merely on skeletal muscle mass and SM‐RA, which might explain the weak‐to‐moderate correlation coefficients found in the current study. Findings of the current study are consistent with the literature in which aerobic fitness was significantly reduced in patients with low skeletal muscle mass index [23, 24, 25]. However, limited research is available that describes the association between aerobic fitness objectively measured with CPET and body composition variables derived from the abdominal CT scan. In a recent study, West and others [16] assessed the association of CT‐scan‐derived body composition with selected CPET variables in patients scheduled for hepatopancreatobiliary and pancreatic surgery. They found that patients with lower SM‐RA values had a statistically significantly lower relative AT (*r* 0.44, *P* < 0.001) and relative VO_2peak_ (*r* 0.57, *P* < 0.001). The current study also found that SM‐RA was significantly correlated with relative AT and relative VO_2peak_ in the univariate analysis (Table 3); however, SM‐RA values were not statistically significantly associated with relative AT and relative VO_2peak_ in the multivariable model (Table 4). Concerning the skeletal muscle mass index, West and others [16] reported a weak association (*r* 0.24, *P* = 0.010) with relative VO_2peak_. Consistent with the current study results, no significant correlation coefficient was found between skeletal muscle mass index and relative AT.

A previous study has shown that patients undergoing major elective colorectal surgery with an AT ≤ 11.1 ml/kg/min have an increased risk for postoperative complications (OR 7.56, 95% CI 4.44–12.86, *P* < 0.001) [8]. Therefore, this study investigated whether a patient with a relative AT ≤ 11.1 ml/kg/min could be predicted from body composition variables derived from the preoperative abdominal CT scan combined with other patient characteristics. A higher ASA score and a higher VAT mass were associated with an increased risk of a relative AT ≤ 11.1 ml/kg/min. However, with an AUC of 0.71, the combination of ASA score and VAT mass had only a moderate ability to discriminate between patients with and without a relative AT ≤ 11.1 ml/kg/min. Nevertheless, this finding suggests that preoperatively assessing body composition from the routinely performed preoperative CT scan, combined with other patient‐related variables, might be useful to enable a preselection of potentially unfit patients, without the need for using additional questionnaires or tests. These potentially unfit (high‐risk) patients should subsequently perform a preoperative CPET to determine the need for a preoperative preventive intervention (e.g., multimodal prehabilitation to improve preoperative aerobic capacity and muscle mass). This preselection might reduce the number of preoperative CPET procedures, thereby saving time and resources.

Preoperative risk assessment is important, as it is the less physically fit patient that will benefit the most from prehabilitation [26, 27]. Despite mounting evidence that prehabilitation has the potential to improve preoperative physical fitness and postoperative outcomes [28, 29], there remains work to be done in order to develop a cost‐effectiveness tool that gives clinicians and policy makers insight into the value of preoperative risk assessment followed by preventive interventions in the right patients. As our results suggest, body composition variables derived from the routinely performed abdominal CT scan, together with other patient characteristics, provides at best limited information on a patient's aerobic fitness. Therefore, the relatively complex and expensive CPET cannot be fully replaced by the preoperative abdominal CT scan. The extent to which other, less sophisticated, tests like the steep ramp test, timed up‐and‐go test, 6‐min walk test and short physical performance battery could refer to preoperative aerobic fitness remains to be evaluated.

The explorative nature of the study, the limited number of patients, and the absence of a prospective sample size calculation are limitations of the present study. Additionally, the fact that only patients with a VSAQ score ≤ 7 METs were referred for CPET might have biased the results, as having all patients perform a CPET prior to colorectal surgery probably would lead to greater accuracy in determining the association between preoperative CT‐scan‐derived body composition variables and preoperative aerobic fitness. These aspects affect statistical analysis and generalizability. Moreover, the studied population is limited to patients undergoing colorectal surgery, who do not necessarily represent the general (surgical) population.

## CONCLUSION

Body composition variables derived from the preoperative CT scan are moderately associated with aerobic fitness as determined from the preoperative CPET. A higher ASA score and a higher VAT mass were associated with an increased risk of a relative AT ≤ 11.1 ml/kg/min as a cut‐off to classify patients scheduled for colorectal surgery as having an increased risk for postoperative morbidity. It seems that the CT scan cannot replace the CPET for preoperative risk assessment on aerobic fitness; however, it may contribute to the (pre)selection of unfit patients.

## ETHICS STATEMENT

Ethical approval for the study protocol (registration number P13‐18) was provided by the Medical Ethics Committee Twente (Dr J.F.F. Lekkerkerker, clinical pharmacologist, chairman) in September 2013.

## PATIENT CONSENT STATEMEN**T**

Written informed consent was obtained from each participant.

## CONFLICT OF INTERESTS

All authors declare that they have no known competing financial interests or personal relationships that could have appeared to influence the work reported in this paper.

## AUTHOR CONTRIBUTIONS

Protocol/project development: AB, NvM, JK, BB. Data collection or management: AB, LvW, DvD, SP, SOD. Data analysis: AB, JvdP, BB. Manuscript writing/editing: AB, LvW, DvD, SP, JvdP, NvM, SOD, JK, BB.

## Data Availability

The data that support the findings of this study are available from the corresponding author upon reasonable request.
